# Dynamic Traffic Signal Control for Isolated Intersections: Enhanced SARSA Reinforcement Learning with Expectation Prediction and Eligibility Traces

**DOI:** 10.3390/s26144367

**Published:** 2026-07-09

**Authors:** Yuhong Gao, Wenyuan Sun, Meiying Jian, Zhaorui Zhang, Zhenying Yan

**Affiliations:** 1 Department of Transportation Engineering, Transportation Institute, Inner Mongolia University, Hohhot 010070, China; swy19980303@outlook.com (W.S.); jianmy@imu.edu.cn (M.J.); 15935790258@163.com (Z.Z.); 2 Inner Mongolia Engineering Research Center for Urban Transportation Data Science and Applications, Hohhot 010070, China

**Keywords:** dynamic traffic control, intelligent signal timing, reinforcement learning, isolated intersections, adaptive signal control, intelligent transportation systems

## Abstract

With the development of intelligent transportation systems, new methods emerge to overcome the limitations of traditional fixed-timing traffic signal control. Among these methods, intelligent signal timing control based on reinforcement learning (RL) boasts promising prospects. Classic RL algorithms have flaws like high estimation bias and lack of robustness, while the newer complex-structure algorithms have issues with training time and unstable convergence speed. To overcome these limitations, this paper develops a two-branch cooperative signal control framework integrating Expected SARSA and SARSA(λ) for isolated signalized intersections, constructing a traffic-adaptive Markov Decision Process (MDP) dynamic decision model using real-time traffic flow data. Expected SARSA reduces policy variance and Q-value overestimation by calculating expected action values. SARSA(λ) adopts eligibility traces for multi-step temporal difference error backpropagation to significantly boost sample utilization efficiency and overall model robustness. Experiments cover algorithm comparison, framework generality and hyperparameter robustness. Experimental results reveal that under the framework proposed in this paper, the two improved algorithms outperform the baseline algorithms in quantitative evaluations conducted from three dimensions. Scenario and parameter tests validate its generality and robustness. This work offers an efficient and reliable solution for real-time adaptive signal control of isolated intersections.

## 1. Introduction

Monitoring data shows that by the end of 2025, China had 395 million motor vehicles, including 302 million automobiles, ranking first globally. Such an enormous vehicle stock has significantly escalated queuing pressure at urban signalized intersections. Corresponding data reveals that the average travel delay of vehicles rose by 8.7 percent year on year, and severely gridlocked intersections may suffer daily economic losses of more than 50,000 yuan. At the same time, road network saturation has evolved into a prevalent worldwide challenge. It further aggravates the imbalance between traffic supply and travel demand, leading to chronic traffic jams, extra carbon emissions, higher energy consumption and rising daily commuting expenses. Moreover, tidal commuter flows together with stochastic fluctuations in real-time traffic volume render conventional fixed-time signal timing strategies unable to adapt flexibly to volatile traffic scenarios. For this reason, traffic congestion alleviation has emerged as a core research priority within the field of intelligent transportation systems. Under such circumstances, rapid progress in deep neural networks has opened up innovative avenues for modern traffic governance. These technologies have been widely adopted in short-term traffic flow forecasting, real-time adaptive signal timing and numerous related research subjects. The adaptive signal control system built upon deep learning and deep reinforcement learning is capable of processing streaming traffic information in real time, continuously lifting the operational efficiency of the whole road network, and providing a reliable technical approach to ease persistent urban traffic congestion.

However, conventional deep learning methods still suffer from key limitations, including value overestimation, poor sample efficiency, and weak robustness under changing traffic conditions. To overcome these issues, this study proposes an improved reinforcement learning framework that enhances policy optimization precision and environmental adaptability for isolated intersection control. The new method establishes an accurate, real-time regulatory model for single-node traffic systems, offering a theoretical and technical foundation for next-generation adaptive traffic control architectures.

### 1.1. Related Work and Motivation

Traffic flow phase transition theory serves as a fundamental theoretical tool to analyze the formation and propagation evolution of urban congestion. It characterizes the abrupt thresholds of three traffic regimes including free flow, synchronized flow and wide moving jams, defines critical boundaries of congestion outbreak at signalized intersections, and provides quantitative theoretical support for proactive signal intervention [1]. Different from traditional two-phase traffic models that merely classify traffic into smooth flow and congestion, the three-phase traffic flow theory clarifies that intersections act as core network bottlenecks. When approaching saturation capacity, inflow traffic triggers an abrupt shift from free flow to synchronized flow without gradual transition. Minor fluctuations in traffic volume can lead to rapid queue accumulation and unbalanced upstream and downstream traffic. Signal timing acts as the core control measure to dynamically adjust critical phase transition boundaries and slow or stop large-scale congestion spread. Scholars keep updating quantitative methods for congestion phase transition boundaries. Existing studies build piecewise macroscopic fundamental diagrams based on three core traffic parameters namely occupancy, flow and speed. Critical saturation flow rate is adopted as the core indicator to recognize phase transitions, enabling real-time judgment and early warning of congestion onset at intersections. Combined with critical slowing-down effects and spatial-temporal graph neural networks, precursor fluctuations of phase transitions can be captured to adjust dynamic signals before vehicle spillover, making up for the defect of passive post-congestion regulation in conventional control strategies. Phase transitions at intersections feature prominent metastability. Long-term traffic volumes hovering around critical ranges can trigger cascading congestion propagation under short-term flow disturbances. Mismatched signal timing across adjacent intersections will aggravate such effects and generate upstream traffic shockwaves, queue spillover and partial network deadlock. Improper green light durations will shift phase transition thresholds and raise congestion risks in multiple travel directions [2].

Based on inherent rules of traffic flow phase transition theory, existing research establishes a systematic analytical framework for urban road congestion evolution, tracing the full life cycle of congestion from three dimensions including supply-demand imbalance, spatiotemporal heterogeneity and ineffective traffic management. Urban congestion falls into recurrent and non-recurrent categories. Recurrent congestion stems from periodic commuter surges and overall supply-demand mismatch during rush hours, as fixed signal timing fails to match time-varying phase transition thresholds. Non-recurrent congestion arises from random disturbances such as traffic collisions, road maintenance and large-scale events, which quickly cross phase transition boundaries and trigger short-term widespread congestion. Both types demand strong real-time adaptive response capacity from signal control systems. Urban congestion shows obvious spatiotemporal differentiation. Once a single intersection crosses the phase transition threshold, congestion shockwaves travel upstream along road segments. Lack of coordinated timing between upstream and downstream intersections will cause successive phase transitions and network-wide congestion. Congestion evolves following a complete sequential chain, and each stage requires distinct optimized signal control targets. Consistent conclusions from systematic review papers reveal a core pain point in current congestion governance. Traditional signal control only optimizes congestion relief in later stages, lacking pre-emptive prevention logic centered on phase transition boundaries, and cannot curb congestion phase transitions at the source [3].

Against this backdrop, deep neural network-based reinforcement learning adaptive signal control (RL-ATSC) has become a leading approach. It treats each intersection as an independent agent that adjusts timing based on real-time traffic dynamics and optimizes policies through continuous interaction. It reduces average waiting time, exploits road capacity, and has low deployment and maintenance costs, thus showing significant engineering value [4].

Current reinforcement learning (RL)-based traffic signal control research still has prominent limitations. Most studies only optimize signal timings for single vehicle types and realize local coordination of adjacent intersections, failing to meet the priority passage demands of buses and emergency vehicles [5]. To solve these problems, some researchers model multi-intersection control as a Markov game and adopt the Multi-Agent Transformer (MAT) architecture to improve network-wide coordinated control performance. Transit Signal Priority (TSP) is a key strategy for improving public transport service and traffic equity, but traditional TSP ignores bus passenger load differences, resulting in suboptimal control effects. To this end, scholars apply Connected Vehicle (CV) technology to acquire real-time bus passenger volume data and construct a Deep Q-Network (DQN)-based signal control model with the optimization objective of minimum per capita delay. Simulations verify that the model reduces delays for both buses and private vehicles and exhibits excellent dynamic adaptability in peak and off-peak traffic scenarios [6]. Wang et al. [7] proposed a Proximal Policy Optimization-based Traffic Signal Control (PPO-TSC) model, which takes vehicle delay and lane queue density as state inputs and designs a targeted reward function. Comparative experiments on the SUMO platform show that the proposed model reduces vehicle travel time by 24%, improves traffic efficiency by 45%, and increases average vehicle speed by 16% compared with traditional signal control methods.

Fan et al. [8] summarized two core deficiencies of existing deep RL signal control algorithms: severe performance degradation of trained agents under sudden changes in turning traffic proportions, and mismatched built-in signal phase switching rules with actual traffic operation. In addition, current lane management research for mixed autonomous vehicle traffic mainly focuses on highways, while few studies explore the joint optimization of lane layout and signal timing at signalized intersections despite their strong coupling. Against this gap, this study constructs a joint optimization framework of dedicated autonomous vehicle lanes and adaptive signal timings for isolated intersections. It adopts an analytical model to calculate intersection saturation flow and vehicle delays under different autonomous vehicle penetration rates, establishes a Mixed Integer Non-Linear Programming (MINLP) model, and designs a decomposition heuristic algorithm for efficient model solving. Numerical simulations and sensitivity analyses validate the effectiveness of the joint optimization scheme and clarify the applicable boundary of dedicated autonomous vehicle lanes for improving intersection efficiency [9].

Q-learning is a classic model-free off-policy RL algorithm. It requires no accurate traffic environment model and obtains optimal control strategies via iterative value iteration. With good adaptability to stochastic traffic changes and delayed reward characteristics, it is widely used in single-intersection signal optimization [10,11,12,13]. However, its high hyperparameter sensitivity and heavy computational overhead limit its application in practical low-latency traffic control scenarios.

To enhance the adaptability of signal control strategies to random traffic disturbances, researchers have extensively optimized deep RL algorithms for single-intersection signal control. For mixed traffic flow, some studies propose a deep RL longitudinal trajectory control method for Connected and Automated Vehicles (CAVs) to balance intersection throughput and driving comfort. A potential function-based structured reward function is designed to ensure driving safety and fuel economy of automated vehicles under signal constraints, with multi-scenario simulations verifying its reliability [14,15]. Recent studies also optimize RL algorithms for multi-objective demands including green traffic and special vehicle priority. Szoke et al. [16] proposed a green intersection control scheme based on successor feature RL, which balances throughput improvement and emission reduction by dynamically adjusting objective weights according to real-time traffic demand, outperforming traditional DQN, heuristic control and classic adaptive timing methods. Wang et al. [17] developed an offline RL model SD3-Light to reduce peak-hour vehicle travel time via dynamic green light duration adjustment. Zheng et al. [18] designed the Pri-DDQN algorithm by integrating prioritized experience replay and Double Q-learning, which balances algorithm exploration and exploitation, and reduces vehicle queue length and travel delay. Lv et al. [19] embedded the ACmix module into Rainbow DQN to build a signal optimization system balancing traffic efficiency and low-carbon goals, which achieves better comprehensive control performance than fixed timing, standard DQN and D3QN models, with promising engineering application value for green transportation.

To address the insufficient utilization of vehicle route information and limited environmental observation of existing algorithms, some studies construct a Soft Actor-Critic (SAC)-based distributed decision model. The model fuses multi-dimensional traffic data including vehicle position, travel route, downstream network capacity and local traffic pressure to improve decision accuracy in partially observable traffic environments, and deploys a weighted real-time priority control mechanism to guarantee special vehicle passage priority. Simulation results show that the scheme effectively shortens vehicle queue length and waiting time, maintains strong robustness against sudden traffic fluctuations, and imposes no obvious negative impact on social vehicle traffic efficiency [20]. Overall, compared with traditional RL algorithms such as Q-learning and vanilla DQN, deep RL algorithms have significant advantages in single-intersection adaptive signal control, capable of optimizing traffic distribution, reducing travel delay and cutting traffic carbon emissions.

Single-intersection signal control ignores the flow coupling between road network intersections, which is inconsistent with actual road network operation. Accordingly, recent research has shifted to centralized road network control and multi-agent collaborative global optimization. Hu et al. [21] built a Deep Deterministic Policy Gradient (DDPG)-based centralized network signal control model, which generates unified signal control commands for all intersections based on global network information. Seven-hour peak-hour network simulations verify its superior control performance and robustness. For large-scale urban road networks, Zhang et al. [22] proposed an Adaptive Value Decomposition Multi-Agent Actor-Critic (AVDMAC) algorithm with parallel training strategies to realize network-wide collaborative traffic control, featuring faster convergence, higher computational efficiency and better global optimization effects. Zhai et al. [23] constructed a Heterogeneous Graph Attention Network and Multi-Agent Reinforcement Learning (HGAT-MARL) framework. The heterogeneous graph accurately depicts diverse road network traffic elements, and multi-agent global information sharing realizes network-wide traffic flow optimization, with measured dataset tests proving its effectiveness in reducing vehicle travel time. Fu et al. [24] proposed a contrastive learning-integrated two-stage CLlight signal control framework, which achieves lower average vehicle travel time and waiting time than mainstream algorithms in public simulation and real road network dataset tests.

Multi-Agent Reinforcement Learning (MARL) is the mainstream technology for multi-intersection collaborative signal control. Nevertheless, most existing algorithms only aggregate adjacent intersection states for strategy optimization and fail to capture deep interactive relationships between agents, restricting further improvement of network-wide coordinated control performance.

Given the complex game relationship between human-driven vehicles and automated vehicles in mixed traffic, integrated signal-vehicle coordinated control has become a cutting-edge research direction. Many studies adopt RL to solve coordinated control problems under mixed traffic and road infrastructure scenarios. Du et al. [25] proposed the Advanced Decision Reinforcement Learning Traffic Signal Control (AD-RLTSC) algorithm. It considers the influence of signal detection range on timing results, introduces Trust Region State (TRS) to optimize decision-making, and equips a dual countdown system for unified control instruction release to human-driven vehicles and CAVs, effectively improving intersection throughput and traffic flow stability [26].

To solve insufficient signal-vehicle interaction and disconnection between prediction and decision-making in connected vehicle environments, researchers developed a Stackelberg Game-based Multi-Agent Reinforcement Learning (SGMRL) integrated vehicle-road control framework. Taking signal controllers as game leaders and automated vehicle fleets as followers, the framework adopts an improved Dueling Double Deep Q-Network (D3QN) to solve game equilibrium. Simulations under different autonomous vehicle penetration rates confirm that the framework significantly reduces vehicle queue length and travel delay [27]. Wang et al. [28] proposed the Shared Experience Multi-Agent Advantage Actor-Critic (SEMA2C) framework to alleviate the sparse reward problem of MARL, which ensures emergency vehicle priority passage without reducing overall network traffic efficiency, outperforming comparable multi-agent algorithms. Li et al. [29] established a Signal-Vehicle Cooperative (SVC) control architecture based on SAC, which synchronously optimizes signal timings and CAV travel trajectories to balance traffic efficiency and driving safety, delivering better control performance than independent signal control or vehicle trajectory regulation strategies.

Moreover, several studies focus on capacity optimization of minor-major road priority intersections. Relying on the communication capabilities of CAVs and ordinary Connected Vehicles (CVs), a capacity model for mixed connected vehicle fleets on minor roads is established. Based on Markov chain theory, intersection capacity is quantitatively evaluated combining headway, lane occupancy duration and lost travel time, and the fleet scale and cooperative driving strategies of two types of connected vehicles are optimized. Simulations verify that the model effectively improves minor-road capacity under variable connected vehicle penetration rates [30].

In addition, emerging adaptive control methods focus on enhancing interpretability, real-time performance and reducing pre-training dependence. Apostol [31] proposed an IoT digital twin traffic signal control method based on tabular Q-learning, which evaluates vehicle waiting time and queue length via auditable tables. Tested on UDOT ATSPM data, the Q-learning agent reduces per-vehicle waiting time by 15.5% and queue length by 12.3% compared with fixed-time and actuated controllers. Shafik and Rakha [32] developed a pre-training-free decentralized Nash bargaining (DNB) adaptive controller without fixed signal cycles, which optimizes each signal phase duration based on real-time traffic density. In tests on two isolated intersections, the DNB controller reduces average delay by up to 54% and queue length by up to 63% versus Webster’s method, with excellent transferability.

These studies together represent major strides in cooperative traffic control via three innovations: formal game-theoretic modeling of agent interactions, redefining controller-vehicle hierarchical roles, and dynamic right-of-way optimization among humans, vehicles, infrastructure. These frameworks offer theoretical insights and practical guidance for developing collaborative, vehicle-infrastructure integrated intelligent transportation systems.

### 1.2. Main Contributions

Existing research has verified that classic reinforcement learning algorithms such as Q-learning and Deep Q-Network (DQN) are capable of optimizing traffic signal control performance. Nevertheless, these algorithms suffer from inherent drawbacks including systematic overestimation bias, training oscillation and insufficient stability. While sophisticated deep reinforcement learning algorithms emerging in recent years can effectively mitigate the aforementioned defects of traditional methods, they introduce new challenges such as slow convergence and lengthy computation time per episode. Reducing training episodes will readily trap agents into local optima and degrade the learning accuracy of control policies. In addition, real-world traffic flows feature random short-term fluctuations, and complex models exhibit weak adaptability to dynamic vehicle arrivals. Within high-dimensional spatiotemporal systems, algorithmic biases propagate through coupling correlations, destabilizing timing strategies and deteriorating road network efficiency. To address the aforementioned challenges, this paper constructs an algorithm framework with fast convergence, high precision and stable training based on well-established lightweight RL models Expected SARSA and SARSA(λ), which adapts to signal control scenarios featuring volatile traffic flows and complex operating conditions.

To address these challenges, this study includes the following research contents:

1. This study constructs a control framework centered on two improved SARSA algorithms, Expected SARSA and SARSA(λ), and applies it to adaptive traffic signal control at isolated intersections to address the issues of value overestimation, low sample efficiency, and insufficient robustness under sudden traffic condition changes in traditional reinforcement learning, while also mitigating the drawbacks of emerging complex RL models such as slow convergence and high computational overhead per iteration.
2. A systematic comparative analysis is conducted between the proposed reinforcement learning-based control model and the Webster-based fixed-time control, Deep Q-Networks (DQN), and emerging complex reinforcement learning algorithms. The evaluation employs a multi-indicator integrated assessment framework, aiming to quantify the optimization performance of each algorithm in a quantitative manner.
3. On the basis of realizing signal control for isolated intersections, the algorithm framework built on Expected SARSA and SARSA(λ) is extended to multiple simulation scenarios of isolated intersections to verify the general adaptability of the proposed framework. Comparative experiments on hyperparameters such as learning rate and exploration rate are also carried out to fully validate the parameter robustness of the model.

The core innovation of this paper lies in constructing a customized integrated architecture, dedicated Markov Decision Process (MDP) modeling scheme and collaborative decision-making mechanism oriented to traffic signal control relying on these two well-established algorithms. The main contributions are summarized as follows:

- This study innovatively constructs a two-branch collaborative integrated framework for adaptive signal control at isolated intersections based on Expected SARSA and SARSA(λ). Unlike existing research that applies the two algorithms separately, this paper designs a shared decision-making kernel equipped with a unified traffic state input interface, a standardized lane pressure reward feedback mechanism and consistent safety constraints for phase switching. Parallel complementary optimization of the two branches is realized to jointly mitigate intersection congestion.
- A dedicated MDP model for traffic scenarios is established for isolated signalized intersections, and a normalized lane pressure reward function independent of road segment length is designed. This customized MDP modeling scheme constitutes the exclusive modeling innovation of this paper, which adapts the two general reinforcement learning algorithms to dynamic and multi-constrained traffic signal control scenarios.
- The inherent strengths of the two existing algorithms are decoupled and complementarily utilized within the unified framework. The built-in expectation prediction mechanism of Expected SARSA is adopted to reduce policy variance and restrain Q-value overestimation, while the eligibility trace multi-step error propagation mechanism inherent in SARSA(λ) is leveraged to improve sample utilization efficiency and accelerate policy convergence. Via two parallel branches, the framework simultaneously optimizes dual objectives including vehicle waiting time and queue length.
- Comprehensive validations are conducted on the SUMO simulation platform from three dimensions including comparison with baseline algorithms, generalization tests on multi-lane intersections, and hyperparameter sensitivity analysis. Experimental results fully demonstrate that the proposed integrated architecture can maintain stable optimization performance under volatile traffic flows and hyperparameter disturbances, offering a lightweight and low-latency practical solution for adaptive signal timing at real-world isolated intersections.

## 2. Methodology

In this section, we introduce the basic knowledge of the traffic signal control (TSC) problem and propose our Improved Reinforcement Learning Method.

### 2.1. Problem Description

#### 2.1.1. Phase

The foundation of the traffic problem lies in the phase, which is the fundamental unit of traffic signal control (TSC), wherein all permitted movements are non-conflicting. A signalized intersection is formally defined as a traffic node comprising ingress arterial segments with configurable lane arrangements. Each intersection is regulated by a discrete set of phases *Φ* = {*φ*_1_, *φ*_2_,…,*φ_n_*}, where each phase φ_k_ ∈ Φ specifies conflict-free path assignments that define permissible movement vectors. Operationally incompatible phases (e.g., φ_1_ and φ_5_) are distinguished by intersecting vehicle trajectories, which impose mutual exclusivity constraints. Modern traffic signal control systems tackle two interdependent optimization challenges: (1) the allocation of cycle duration (temporal granularity) and (2) the coordination of phase sequences (spatial efficiency). This study adheres to empirically validated signal timing boundaries [t_min_, t_max_] and employs Markovian phase-switching control through state-aware reinforcement learning. By integrating these two optimization layers, the framework enables dynamic flow regulation via adaptive conflict resolution, thereby effectively curbing the propagation of congestion caused by saturation.

By using DRL, we regard the TSC problem as Markov Decision Process (MDP). An individual DRL agent controls one of the intersections.

#### 2.1.2. State

The operational state of the intersection is described by a set of multidimensional parameters, including:

1. Real-time vehicle accumulation metrics, which quantify the number of vehicles across approach lanes;
2. Queue propagation characteristics at entry points, capturing the formation and dissipation of queues;
3. The current temporal status of traffic signal phases, reflecting elapsed time within the active phase.

#### 2.1.3. Action

The control agent executes decisions either to extend the current phase or to initiate a phase transition. The dimensionality of the action space is proportional to the number of phases in the intersection’s signal plan. Specifically, the action space is formulated as follows. Action space: A = {0,1}, which is a binary discrete action space. The meanings of the actions are defined as follows: a = 0 means maintaining the current signal phase unchanged; a = 1 means switching to the immediate next phase in the phase set according to the preset phase sequence, with a 3-s amber time inserted during the switching process. Therefore, the action does not directly select a specific phase (such as north-south straight movement, east-west left turn, etc.), but determines whether to switch to the next preset phase on the basis of the current phase. This is a binary decision action space with a fixed phase sequence.

#### 2.1.4. Reward

This study adopts a reward generation mechanism based on lane-normalized pressure, and takes the pressure difference among different phases at the intersection as the core feedback signal for the reinforcement learning strategy. Lane pressure reflects the real-time traffic flow density, while the phase pressure difference quantifies the spatial imbalance degree of upstream and downstream lanes. To eliminate the influence of the physical length of different road segments on pressure evaluation, normalized pressure considering road capacity is introduced.

Let q be the queue length on a lane, and $Q_{\max}$ the maximum capacity (corresponding to queue length reaching the road length L). The normalized pressure p is defined as:

(1) $$p=\frac{e^{kq}-1}{e^{kQ_{\max}}-1}$$

where the coefficient k is determined by the boundary condition p=1 when $q=Q_{\max}$:

(2) $$k=\frac{\ln(2)}{Q_{\max}}$$

Substituting gives the final expression:

(3) $$p=2^{q/Q_{\max}}-1$$

This design yields approximately linear growth at low queue lengths and exponential growth at high queue lengths, with values in [0, 1].

For a given phase ϕ with an entering lane in and an exiting lane out, the pressure difference is:

(4) $$\Delta p_{\phi }=p_{in}-p_{out}$$

The total pressure at intersection n is the sum of pressure differences over all phases:

(5) $$P_{n}=\sum_{\phi }\Delta p_{\phi }$$

The reward function takes the negative value of the intersection pressure to encourage the agent to reduce overall pressure:

(6) $$R_{n}=-P_{n}$$

A more negative reward indicates higher intersection pressure, prompting the agent to adopt signal timing strategies that balance traffic across all approaches. In the multi-agent cooperative framework, each intersection computes its own reward independently while sharing neighborhood state information to achieve global optimization of the regional road network.

### 2.2. Model Construction

The study outlines an operational architecture for integrating reinforcement learning (RL) into traffic control systems. The RL agent determines optimal control actions through computational optimization and issues operational directives for traffic signal phase transitions. These directives are first sent to environmental controllers, which forward them to middleware systems. Within the middleware, the commands undergo protocol encapsulation and are converted into standardized communication packets, which are then transmitted to Traffic Signal Light Units (TSLUs) via a REST API.

The processing module in the TSLU evaluates each phase transition request against predefined operational constraints. Based on this evaluation, it either maintains the current phase or executes a phase transition. A structured response including operational parameters like verified phase configuration and signal state metadata transmits back via middleware. Environmental controllers subsequently perform state mapping to synchronize the simulated traffic light states with the physical system’s configurations.

**Figure 1:**
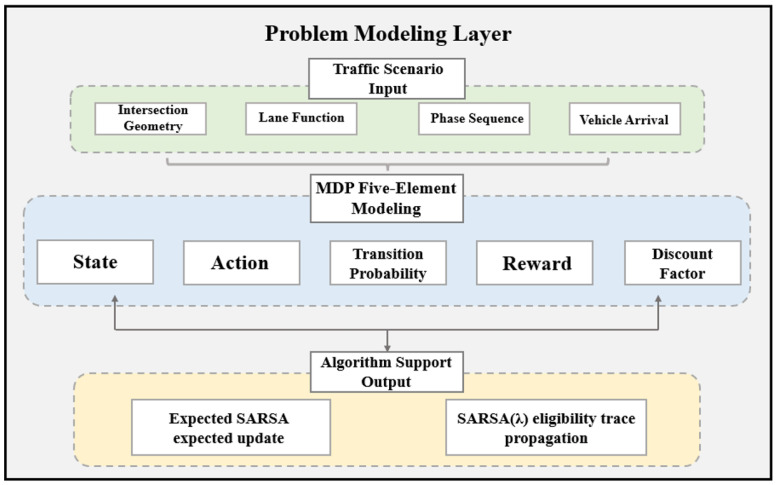
Closed-loop operation architecture of reinforcement learning adaptive traffic signal control system.

Based on the above MDP modeling, to enable effective control of actual traffic signal systems by reinforcement learning policies, it is necessary to construct a closed-loop operational framework that covers environmental perception, decision optimization, and instruction execution. This framework must not only ensure the real-time performance and reliability of phase-switching commands, but also support continuous interaction between the agent and the traffic environment, so as to continually adjust and optimize the control policy. Moreover, to accommodate the multi-tiered and multi-module distributed deployment requirements of practical traffic management systems, the framework needs to clearly define the division of responsibilities and communication protocols among functional components. To this end, this study designs the closed-loop operational architecture shown in Figure 1, which clearly illustrates the information flow relationships and control command flow paths among the reinforcement learning agent, environmental controllers, middleware, and traffic signal light units (TSLUs), thereby providing a system-level implementation blueprint for the deployment and validation of subsequent algorithms in simulation environments.

### 2.3. Algorithm Proposal

SARSA as a temporal-difference reinforcement learning method, is applicable to policy evaluation and control within Markov Decision Process (MDP) environments. It adheres to the on-policy paradigm, updating the action-value function according to the current behavioral policy to ensure coherence between action selection and value estimation. The algorithm commonly incorporates ε-greedy exploration to balance exploration and exploitation. This foundation fundamentally distinguishes SARSA from off-policy algorithms such as Q-learning, which optimizes Q-values by maximizing estimated future rewards without considering the actual implemented policy. In contrast, SARSA fully takes policy stochasticity into account, which helps alleviate Q-value overestimation and improves learning stability.

**Figure 2:**
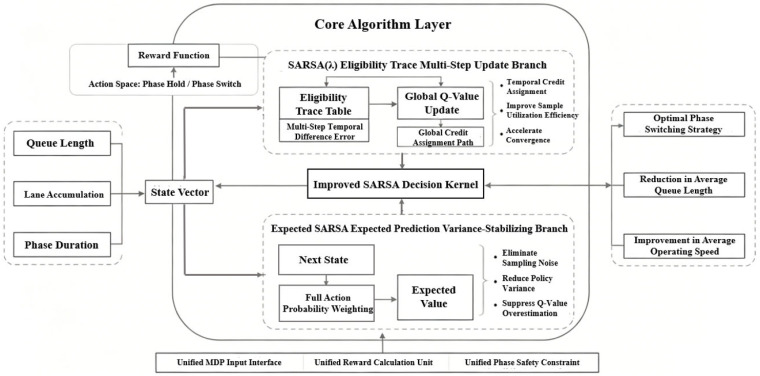
Dual-path optimization framework of improved SARSA algorithm for isolated intersection signal control.

However, when applied to isolated intersection signal control, SARSA exhibits inadequate convergence speed, significant training variance, and limited dynamic adaptability. To overcome these issues, we propose a dual-path enhanced architecture that combines the multi-step backward updates of SARSA(λ) with the expected value optimization of Expected SARSA, and employs a phased approach to enhance control performance. As shown in Figure 2, this framework includes two independent optimization branches.

The SARSA(λ) branch utilizes multi-step temporal-difference error backpropagation to improve sample utilization and accelerate convergence, aiming to reduce average queue length.

The Expected SARSA branch eliminates Q-value overestimation by probability-weighted averaging over all actions, suppresses sampling noise and policy variance, and optimizes average waiting time and travel speed.

The two branches achieve joint optimization of signal control decisions through distinct mechanisms and approaches. The specific implementation logic is as follows. During the decision-making phase, the two parallel branches perform algorithm calculations synchronously and share the shared decision kernel constructed in this paper. Meanwhile, a unified traffic state input interface is adopted together with a standardized lane pressure reward feedback function, and identical safety constraints for phase switching are imposed. Such settings realize parallel collaboration and complementary merits of the two branches for optimization. Finally, two groups of experimental results dominated by the Expected SARSA branch and the SARSA(λ) branch are output separately.

#### 2.3.1. SARSA(λ) Algorithm

SARSA(λ) combines the standard SARSA algorithm with eligibility traces to accelerate learning and improve the reuse of past experiences. By distributing temporal-difference error (TD-error), across multiple state-action pairs, SARSA(λ) alleviates the slow learning characteristic of vanilla SARSA, effectively bridging Monte Carlo and temporal-difference learning [33].

The algorithm initializes the state-action value function, usually assigned zero or small random values, alongside an eligibility trace table that starts at zero. The agent begins by observing the initial state and selecting an initial action. At each time step, it executes the action, observes the reward and next state, selects the next action, and then computes the TD-error using the following formula:

(7) $$\delta_{t}=r+\gamma Q(s^{\prime },a^{\prime })-Q(s,a)$$

Here, γ is the discount factor, used to measure the importance of future rewards. Q(s,a) is the estimated value of the current state-action pair, and $Q(s^{\prime },a^{\prime })$ is the estimated value of the next state-action pair. And update the eligibility traces:

(8) E(s,a)=E(s,a)+1

For all state-action pairs, update (s,a):

(9) $$Q(s,a)\leftarrow Q(s,a)+\alpha \delta_{t}E(s,a)$$

(10) E(s,a)←γλE(s,a)

α is the learning rate, which controls the step size of the updates. λ is the eligibility trace decay factor, which controls the importance of historical information. Update the current state and action to the next state and action $s\to s^{\prime },a\to a^{\prime }.$ We define the TD error as:

(11) $$\delta_{t}=r_{t}+\gamma Q(s_{t+1},a_{t+1})-Q(s_{t},a_{t})$$

The combination of eligibility traces and TD-error forms the update rule for SARSA(λ):

(12) $$Q(s,a)\leftarrow Q(s,a)+\alpha \delta_{t}E_{t}(s,a)$$

The recursive update for the eligibility trace $E_{t}(s,a)$ is:

(13) $$E_{t}(s,a)=\gamma \lambda E_{t-1}(s,a)+1(s,a=s_{t},a_{t})$$

When λ = 0, SARSA(λ) degenerates to SARSA; when λ = 1, SARSA(λ) approaches the Monte Carlo method, using complete information from the entire episode.

#### 2.3.2. Expected SARSA Algorithm

Expected SARSA is an extended version of the SARSA algorithm. Different from SARSA, which updates Q-values using only a single action sampled under the current policy, Expected SARSA computes the expected value over all available actions in the next state. This adjustment greatly stabilizes the learning process. By leveraging the expectation of the action-value function for the subsequent state, Expected SARSA lowers variance and mitigates its sensitivity to the randomness of the ε-greedy exploration strategy [34].

The algorithm operates as follows: It initializes the state-action value function Q(s,a) (typically setting all values to 0). The agent observes the initial state *s* and selecting the initial action *a*. At each time step *t*, the agent executes the selected action, observes the reward *r* and the next state $s^{\prime }$, and then calculates the expected value for the next state.

(14) $$E[Q(s^{\prime },a^{\prime })]=\sum_{a^{\prime }}\pi (a^{\prime }|s^{\prime })Q(s^{\prime },a^{\prime })$$

Here, $E[Q(s^{\prime },a^{\prime })]$ represents the expected value calculation, replacing the maximum value computation used in SARSA(λ). $\pi (a^{\prime }|s^{\prime })$ denotes the probability of taking action $a^{\prime }$ under the current policy at the next state $s^{\prime }$. Compute the TD-Error:

(15) $$\delta_{t}=r+\gamma E[Q(s^{\prime },a^{\prime })]-Q(s,a)$$

Update the Q-value:

(16) $$Q(s,a)\leftarrow Q(s,a)+\alpha \delta_{t}$$

Update the current state and action to the next state and action $s\leftarrow s^{\prime },a\leftarrow a^{\prime }.$

## 3. Interactive Simulation Implementation

Modern intelligent transportation simulation systems typically employ a client-server architecture to decouple the simulation engine from the control logic. This setup generally includes a simulation server running SUMO and a control client implemented in Python 3.9, which are connected via the Traffic Control Interface (TraCI) protocol for real-time data exchange and control.

The TraCI interface library enables bidirectional data exchange, allowing for programmatic access to traffic simulation parameters through three core operational capabilities:

1. Acquisition of Microscopic Traffic State Variables: This includes obtaining information on vehicular positioning and kinematic profiles.
2. Dynamic Reconfiguration of Traffic Signal Phase-Timing Parameters: It permits real-time adjustments to the timing of traffic signal phases.
3. Real-Time Querying of Transportation Network Topological Configurations: This capability facilitates the retrieval of up-to-date information about the topological structure of the transportation network.

**Figure 3:**
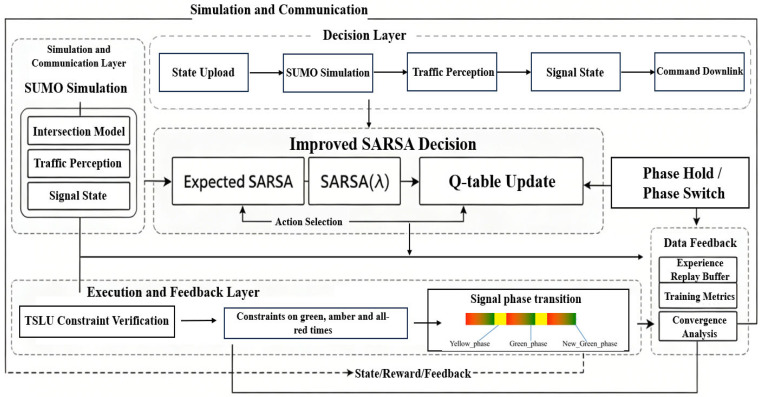
MDP closed-loop decision flow of single-intersection adaptive signal control based on reinforcement learning.

The simulation server performs high-fidelity modeling of traffic dynamics, encompassing vehicle motion simulation, signal state transitions, and network topology calculations; the control client incorporates a reinforcement learning-based decision engine and relies on the closed-loop paradigm of “environmental perception, policy decision, and control actuation” to achieve intelligent signal optimization. At the optimization level, the decision framework operates under multi-objective criteria, and the reward function is designed to simultaneously pursue three performance metrics: accelerating Q-value convergence, reducing intersection delays, and enhancing network throughput. Figure 3 illustrates the complete MDP closed-loop decision flow for single-intersection adaptive signal control based on reinforcement learning. Specifically, the system first extracts multi-dimensional traffic state vectors, including lane queue length, residual phase time, and real-time vehicle speed, from SUMO via TraCI as MDP state inputs; the improved SARSA agent then outputs binary phase-switch actions according to the current policy; subsequently, the reward function calculates the normalized lane pressure difference to provide feedback signals; and this entire process iterates with each simulation step, continuously updating the Q-table and progressively optimizing signal timing strategies.

The adaptive traffic signal control framework for isolated intersections was implemented using a Python-based computational platform. This system incorporates advanced reinforcement learning architectures to optimize the scheduling of signal phase transitions. A formal description of the control algorithm is provided in Table 1.

## 4. Case Study

### 4.1. Experiment Settings

Experimental validation is conducted using a standardized bidirectional four-lane intersection configuration. Within this environment, a comparative performance evaluation is carried out among heterogeneous traffic signal control agents over 5000 simulation steps. Performance metrics are sampled every 5 episodes to facilitate systematic comparison.

The quantitative assessment encompasses the following three dimensions:

1. Policy Optimization Progress: Quantitatively evaluated through the dynamic evolution curves of agent Q-values. The continuous variation of these Q-value curves intuitively reflects the convergence speed and stability of the learned decision-making policy, as well as the overall trend of progressive optimization of the intelligent signal control policy over time.
2. Vehicular Congestion Severity: It is obtained by measuring the average queue waiting time of vehicles at isolated intersections. A longer waiting time indicates greater vehicle accumulation at the stop line, which clearly quantifies the extent of vehicle stacking and queueing pressure across different approaches.
3. Operational Efficiency: The overall traffic operational efficiency at intersections is measured by the average vehicle speed, which directly indicates the fluidity of real-time traffic and reflects the intersection’s overall passage efficiency under the optimized signal timing strategy.

Expected SARSA and SARSA(λ) are rigorously benchmarked against a suite of reinforcement learning baselines. Their overall performance for intersection signal control is comprehensively evaluated using three quantitative metrics, with particular emphasis on model convergence behaviors and traffic throughput at signalized intersections. For the selection of comparative algorithms, the Webster-based fixed-time control strategy and classic DQN are established as fundamental baselines. The SARSA and the structurally complex Rainbow DQN are further introduced to highlight the gains achieved by the proposed framework in sample efficiency and iterative convergence rate.

To ensure experimental integrity amid the inherent parametric sensitivity of DRL models, all algorithms are implemented with standardized hyperparameters. Training procedures are conducted under identical computational conditions. The full results of the comparative analysis are systematically summarized in Table 2.

*lr* represents the learning rate, which is the rate at which the agent updates its policy or value function in each reinforcement learning algorithm. *γ* is the discount factor used to obtain reward values. It determines the present value of future rewards. Λ is a hyperparameter in the Eligibility Trace method. It is employed to control the “memory” length during the learning process. Specifically, λ is a hyperparameter in the Eligibility Trace method. It is employed to control the “memory” length during the learning process. Specifically, λ governs how future rewards are attributed. When λ = 0, only the reward at the current time step is taken into account. The closer λ is to 1, the more the model relies on past rewards, and thus, it depends more on long—term historical information. ε is a hyperparameter in the ε-greedy strategy. Typically, *ϵ* gradually decreases during the training process. This encourages the agent to explore more in the early stages of training and exploit the learned knowledge more in the later stages, epsilon with a default value of 0.05. This means that the agent has a 5% probability of choosing a random action and selects the currently best—known action the remaining 95% of the time.

### 4.2. Simulation Execution

The simulation platform used in this work is Simulation of Urban Mobility (SUMO), which is one of the most widely used open-source microscopic traffic simulators. Our model design and implementation are based on FLOW, which provides DRL-related API to work with SUMO dynamically.

**Figure 4:**
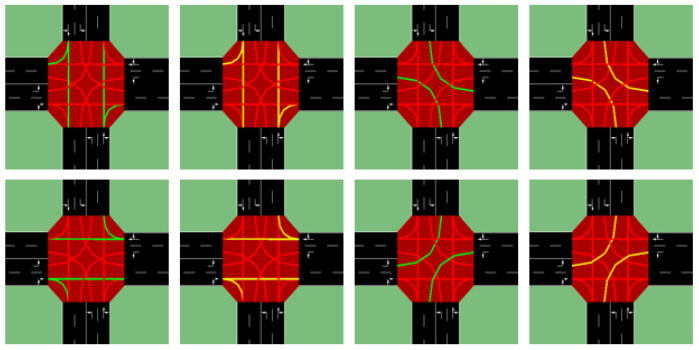
Dynamic interaction between deep learning based on enhanced algorithms and intersections in the SUMO environment.

A four-lane, two-way intersection is established in SUMO, and an enhanced deep learning-based traffic signal control system is dynamically integrated with the SUMO simulation tool. The system adaptively selects signal phases and green light durations based on real-time intersection conditions to achieve adaptive control of vehicles, thereby improving traffic efficiency. A dynamic simulation example of this implementation in SUMO is illustrated in Figure 4.

When the control agent proposes a phase change that differs from the current phase, the Traffic Signal Control Unit (TSLU) performs a safety validation check. The system only allows phase switches that comply with the Minimum Pedestrian Green Interval (MPGI) requirements, which typically align with the minimum green time in the signal timing plan. Any request that shortens the MPGI is treated as a safety risk. Within the reinforcement learning framework, such risk typically arises from the agent potentially terminating the pedestrian green phase prematurely to optimize vehicular traffic efficiency, thereby violating the hard safety constraint for pedestrian crossing. In such cases, the TSLU enforces a phase retention protocol: it automatically rejects the phase change command and maintains the current phase to ensure full regulatory compliance.

The design of optimal phase transition strategies is a complex challenge in traffic engineering. It involves determining the switching times between conflicting traffic flows, such as intergreen intervals and yellow times, and must account for the time required for safe pedestrian crossing, intersection clearing, and signal system preparation for the next phase. These timing parameters depend on various operational factors, such as speed limits, road user types, as well as intersection geometry and layout. Through comprehensive analysis, all runtime constraints in this simulation are summarized in Table 3.

### 4.3. Results Analysis

In reinforcement learning, the Q-value serves as a fundamental metric for evaluating action utility and guiding policy optimization. The Q-function, as a core component of reinforcement learning algorithms, operates within the Markov Decision Process (MDP) framework and iteratively refines action-value estimates through environmental interaction, asymptotically approaching the optimal policy. The agent makes control decisions by selecting actions that maximize cumulative discounted rewards.

In the context of isolated intersection signal control, the evolution curve of Q-values intuitively reflects the agent’s learning process: the convergence characteristics of Q-values not only indicate the agent’s progress toward an optimal phase policy under varying traffic conditions, but also demonstrate the iterative process of the agent shifting from exploration to exploitation, a transition that aligns with the exploration and exploitation tradeoff theory in RL.

The comparative experiments adopt the following groups of control schemes: two branch algorithms (Expected SARSA and SARSA(λ)) derived from the proposed framework whose output results are denoted as Expected SARSA and SARSA(λ) in the subsequent content for differentiation, a Webster-method-based fixed-time signal control strategy acting as the baseline, the classic DQN signal control algorithm, the vanilla SARSA signal control algorithm, and the signal control scheme developed on Rainbow DQN, a prevalent composite deep reinforcement learning model. All simulation experiments are implemented on the standardized microscopic traffic simulation platform constructed in this study, and an isolated two-way four-lane signalized intersection with conventional traffic demand is chosen as the test scenario.

**Figure 5:**
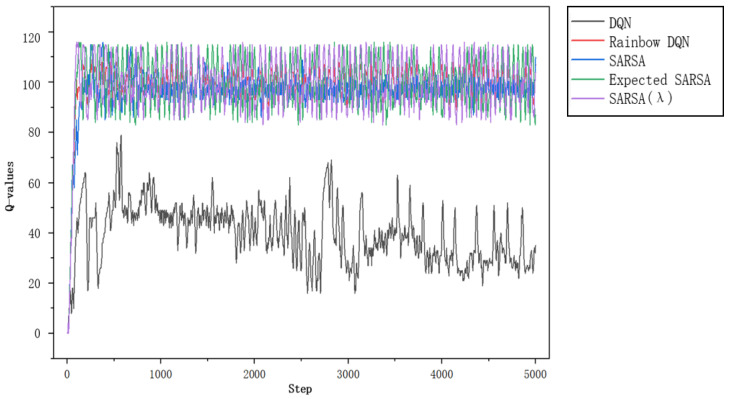
Q-values of various RL algorithms.

Figure 5 presents the comparison curves of Q-values for all algorithms, which intuitively characterize the convergence rate and training stability of different learning strategies and clearly reveal the evolutionary law of intelligent signal control strategies continuously optimized with iterative training.

Quantitative analysis of the mean Q-value trajectories across different reinforcement learning algorithms, as summarized in Table 4, reveals significant performance discrepancies among algorithmic variants. At a significance level of p<0.05, Expected SARSA and SARSA(λ) achieve mean Q-values of 99.608 and 99.413, respectively, corresponding to improvements of 3.2% and 3.0% over the baseline SARSA (96.535). Moreover, compared with the more sophisticated Rainbow DQN (97.322), the two proposed algorithms obtain performance gains of approximately 2.35% and 2.15%, respectively. According to the above experimental results, the improved algorithm proposed in this paper accelerates policy convergence and approaches the optimal signal timing strategy for intersections faster compared with the traditional SARSA. Its control performance also outperforms the Rainbow DQN model with a more complex network structure. The improved algorithm achieves prominent advantages in both convergence speed and single-step computation time, delivering superior overall operational efficiency. It fully verifies that the introduction of the expectation prediction mechanism and eligibility traces can effectively boost model learning efficiency and stabilize the training process.

**Figure 6:**
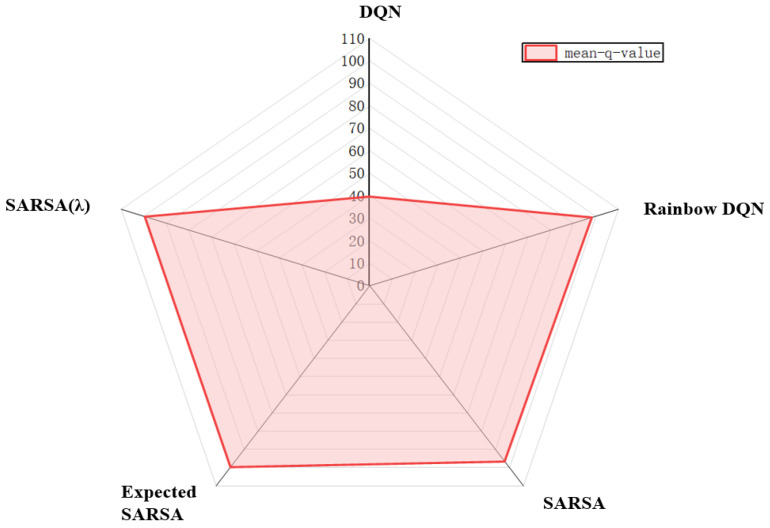
Proportion of Q-values for each reinforcement learning algorithm.

As shown in Figure 6, the Q-value distribution of different reinforcement learning implementations reveals that improved algorithms outperform other methods in searching for optimal control policies, especially for traffic signal phase switching in intersection management.

Expected SARSA replaces the maximum operator with an expectation over stochastic rewards, thereby reducing update variance through probabilistic future-state valuation.

SARSA(λ) introduces a temporal credit assignment mechanism via eligibility traces, which effectively mitigates the issue of delayed rewards in phased control systems.

These modifications allow the learning agent to converge more rapidly toward highly efficient phase-switching strategies under stochastic traffic conditions. Such capabilities are of significant practical value for intelligent transportation systems that require real-time responsiveness and strategic robustness.

Vehicular queueing delay at signalized intersections is a key metric for assessing the performance of traffic control systems. The temporal dynamics of this delay provide direct empirical insight into a reinforcement learning agent’s ability to optimize signal timing strategies.

From the perspective of learning behavior, consistently high average queueing durations suggest the presence of suboptimal phase allocation. This often results from unbalanced phase timing, which can cause localized congestion and lead to a buildup in traffic density. In contrast, a steady reduction in vehicular delays reflects the successful development of a state-action value function within the control agent. By adaptively adjusting the duration and sequence of green phases, the learning mechanism achieves a form of multi-objective Pareto optimization in traffic flow distribution. This capability is especially valuable for urban mobility systems that require a balance between operational efficiency and sustainable infrastructure use.

**Figure 7:**
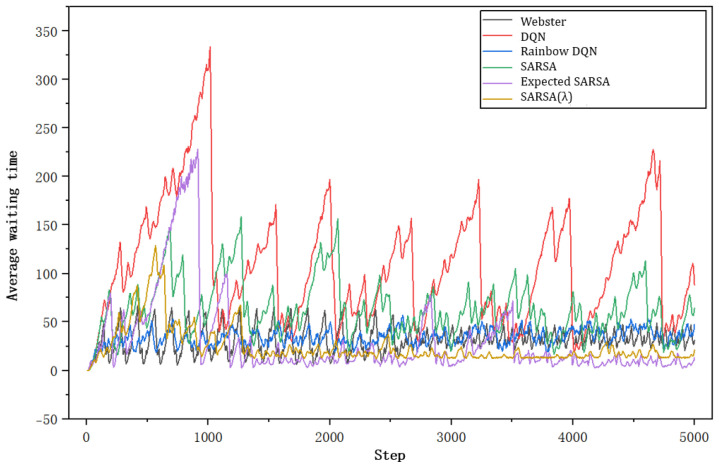
Average vehicle waiting time.

As shown in Figure 7, the evolution of mean vehicular delay under identical training iterations varies significantly among reinforcement learning algorithms. Fixed-time signal control remains stable throughout and acts as the baseline benchmark. In the early training phase, all algorithms exhibit high delay values with severe random fluctuations, reflecting an exploration-dominated stage where agents have limited knowledge of traffic dynamics and frequently select suboptimal actions. As training advances, delays gradually decrease and stabilize, though convergence characteristics differ.

After 4000 training steps, nearly all algorithms reach a plateau, and the proposed algorithm achieves the best performance. Expected SARSA, leveraging expectation-based update mechanisms within the temporal difference framework, better balances exploration and exploitation, thereby accelerating the stabilization process. SARSA(λ), using eligibility traces to address temporal credit assignment, further improves learning efficiency, shortens training time, and enhances parameter stability. Quantitative results from the final training stage confirm that our proposed method yields superior asymptotic performance, with statistically significant reduction in average vehicle delay. These findings demonstrate its effectiveness in generating signal timing strategies that effectively mitigate intersection congestion and optimize overall traffic flow.

**Figure 8:**
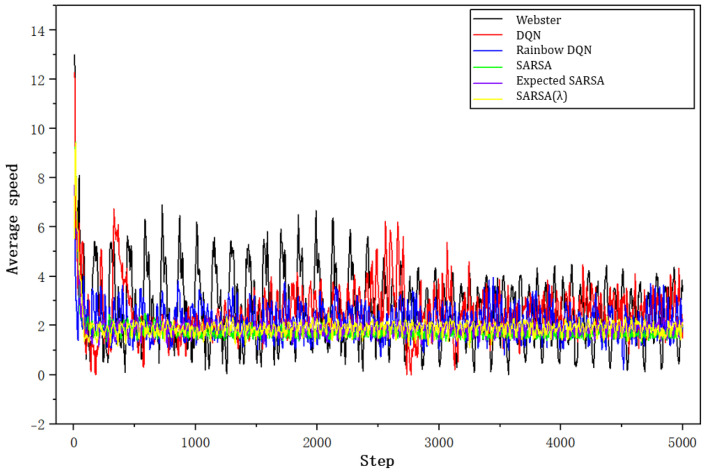
Average vehicle speed at the intersection.

Figure 8 presents the temporal velocity profiles of all algorithms throughout the training process, providing an intuitive comparison of their capabilities in coordinating traffic flow at intersections.

In the early training stages, all algorithms exhibit high average velocities with substantial fluctuations, reflecting the interference of extensive exploration on traffic coordination efficiency. As policy parameters gradually converge, the velocities continuously decrease, indicating that the adaptive signal control strategy is progressively optimized and effectively alleviates traffic congestion. Compared with baseline algorithms, SARSA(λ) shows the most significant convergence effect, achieving not only the lowest velocity but also minimal fluctuations; DQN maintains a relatively high velocity baseline with persistent oscillations, suggesting slower policy stabilization; standard SARSA and Expected SARSA perform at an intermediate level, overall inferior to SARSA(λ). These results validate the advantage of the SARSA(λ) algorithm in the proposed framework, which leverages the eligibility trace mechanism to generate superior phase coordination schemes, effectively regulate vehicle speeds, and comprehensively enhance intersection operational efficiency.

**Figure 9:**
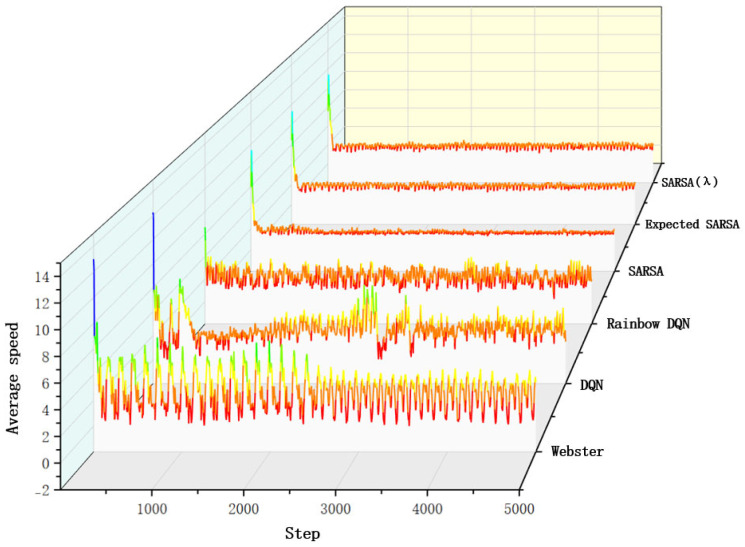
3D plot of average vehicle speed at the intersection.

Figure 9 shows that velocity profiles are closely linked to intersection flow continuity. Higher speeds suggest better vehicular throughput, but may also raise safety concerns by increasing collision risks. In contrast, lower speeds are associated with greater flow stability, reflecting the control agent’s ability to use adaptive signal phasing in response to real-time traffic. This supports improved queue management and smoother vehicle progression.

To interpret intersection operational evolution under the proposed framework, we examine queue length, pressure difference, and waiting time across training stages. In the early stage, exploratory behavior causes frequent phase switching and high waiting time variance. In the mid stage, the agent learns effective transition patterns: queue length decreases and pressure differences balance, improving responsiveness to traffic asymmetries. In the late stage, the intersection reaches stable operation, with low waiting time and smooth phase adaptation. These results demonstrate a smooth transition from congested, unstable conditions to near-optimal adaptive control.

### 4.4. Robustness Analysis

Although the above simulations have verified the performance of the improved SARSA algorithm for signal control at standard isolated intersections, intersections in urban road networks adopt diverse lane layouts and phase configurations. To verify the robustness of the proposed method, simulation environments of isolated intersections with different lane quantities and traffic flows are built for testing, while other relevant variables such as constraint parameters and phase configurations are kept unchanged. In addition, the two core hyperparameters, learning rate and exploration rate, are adjusted to further examine the parameter stability of the algorithm.

#### 4.4.1. Analysis of Different Intersections

**Figure 10:**
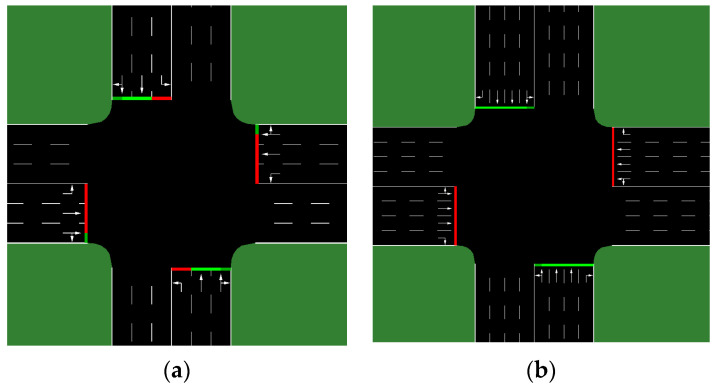
Simulated intersection: (**a**) two-way six-lane; (**b**) two-way eight-lane.

Taking the two-way four-lane intersection as the baseline simulation scenario, two additional intersection models, namely the two-way six-lane intersection and two-way eight-lane intersection, are established. The geometric structure and lane layout of each intersection are illustrated in Figure 10. The two newly added simulation scenarios adopt exactly the same signal phase scheme as the baseline model, while the inflow traffic volume is increased by 50% and 100% respectively to reproduce the high traffic demand at intersections during peak hours.

Three core indicators are selected for evaluation, including the average vehicle queue length at intersections, average vehicle waiting delay, and average link travel speed. The comparative algorithms cover the two improved SARSA algorithms derived from the proposed framework and the vanilla standard SARSA algorithm, so as to quantitatively visualize the performance improvement of the two improved algorithms over the original SARSA algorithm. All reinforcement learning hyperparameters for training and constraint conditions such as green light durations of signal phases are consistent with the configurations presented in the previous sections throughout the simulation.

**Figure 11:**
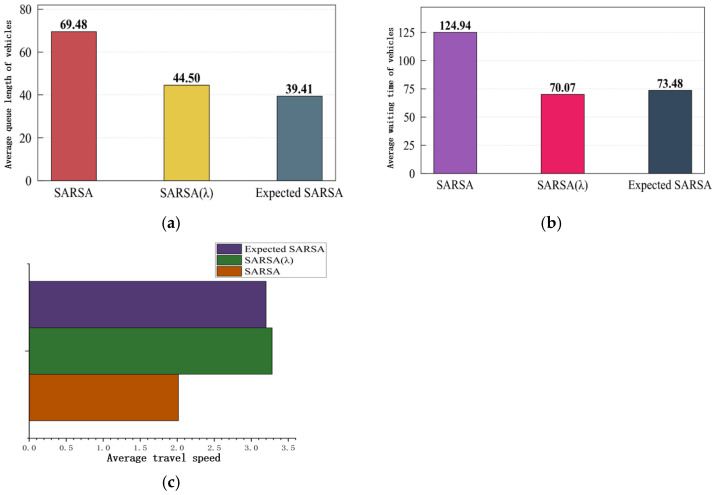
Experimental results at a two-way six-lane intersection: (**a**) Average queue length of vehicles; (**b**) Average waiting time of vehicles; (**c**) Average travel speed.

Multiple repeated experiments are conducted to obtain the average values. The experimental results of three core indicators, i.e., average vehicle queue length, average vehicle delay and average link travel speed for the two-way six-lane intersection, are summarized in Table 5, and Figure 11 visually presents the performance comparison between the two improved algorithm branches and the vanilla SARSA algorithm.

**Figure 12:**
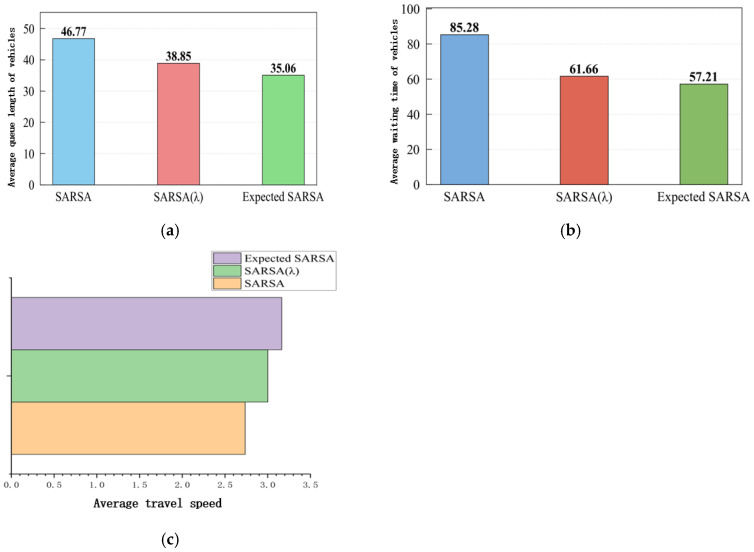
Experimental results at a two-way eight-lane intersection: (**a**) Average queue length of vehicles; (**b**) Average waiting time of vehicles; (**c**) Average travel speed.

Consistent with the experimental configurations and evaluation system specified above, the experimental data of the three core traffic indicators under the two-way eight-lane intersection scenario are summarized in Table 6, and Figure 12 intuitively compares the operational performance of the two improved algorithms and the vanilla SARSA algorithm.

The observed experimental results indicate that, in the two-way six-lane scenario, the average queue lengths of Expected SARSA and SARSA(λ) are reduced to 44.50 m and 39.41 m, respectively; the average waiting times are reduced to 70.07 s and 73.48 s, respectively; and the average travel speeds are increased to 3.282 m/s and 3.200 m/s, respectively. Although the waiting time of SARSA(λ) is slightly higher than that of Expected SARSA, both improved algorithms clearly outperform the original SARSA; moreover, Expected SARSA exhibits a better performance in terms of speed improvement. In the two-way eight-lane scenario, both enhanced algorithms also significantly outperform the original SARSA, with SARSA(λ) maintaining the optimal performance in queue length and waiting time, while Expected SARSA shows a more prominent advantage in speed enhancement, thus complementing each other. Overall, variations in the number of lanes and traffic flow do not substantially weaken the ability of the two improved algorithms to shorten queues, reduce waiting time, and increase travel speed, which fully demonstrates their excellent robustness and generalization capability.

#### 4.4.2. Parameter Sensitivity Analysis

In this section, multiple sets of tuning experiments are carried out for two core hyperparameters, namely the learning rate (*lr*) and exploration rate (ε), to comprehensively verify the robustness of the algorithm proposed in this paper. The groups with *lr* = 0.001 and ε = 0.05 are set as the baseline control groups. Average vehicle queue length and average vehicle waiting time are selected as quantitative evaluation indicators to quantitatively analyze the differential influence of varying hyperparameter values on the model convergence characteristics and real-time signal control performance of intersections. The complete experimental data and statistical results of indicators are presented in Table 7 and Table 8. To strictly control experimental variables, all simulation experiments in this chapter adopt the two-way four-lane isolated intersection proposed in this paper as the unified experimental scenario, and all scene parameters and configurations are completely consistent with the previous settings.

With the exploration rate fixed at ε = 0.05, when the learning rate increases from 0.001 to 0.1, the average waiting time of vanilla SARSA rises from 85.21 s to 130.55 s, and the average queue length grows from 95.72 m to 129.14 m with drastic fluctuations. In contrast, the waiting time of SARSA(λ) and Expected SARSA only increases to 85.20 s and 83.78 s respectively, while their queue lengths merely reach 74.63 m and 76.32 m. The growth amplitudes are far lower than those of vanilla SARSA, and their performance remains significantly better than the baseline throughout the process. The two improved algorithms are insensitive to variations in the learning rate, among which Expected SARSA achieves the optimal stability. This demonstrates its strong hyperparameter robustness, enabling it to maintain efficient control performance without elaborate parameter tuning.

With the learning rate fixed at *lr* = 0.001, the performance of all algorithms improves as the exploration rate rises from 0.05 to 0.25. The waiting time and queue length of vanilla SARSA drop to 76.31 s and 78.94 m respectively; those of SARSA(λ) decrease to 54.81 s and 51.15 m; Expected SARSA achieves the optimal results with values of 53.07 s and 52.45 m. The two improved algorithms significantly outperform vanilla SARSA under all exploration rate settings, and the performance gap narrows as the exploration rate increases. This verifies their favorable adaptability to changes in the exploration rate, and precise parameter adjustment is unnecessary in practical applications.

## 5. Conclusions

With the development of intelligent transportation systems, traditional fixed-timing signal control fails to cope with stochastic traffic fluctuations and supply-demand imbalances. Adaptive reinforcement learning provides a promising technical route, yet the classic SARSA algorithm is plagued by large action-value estimation bias, unstable training performance and insufficient adaptability to dynamic traffic conditions. Meanwhile, sophisticated deep reinforcement learning architectures proposed in recent years suffer from slow convergence, high computational overhead per iteration and weak generalization under sudden traffic flow variations. To tackle the above dual drawbacks, this paper integrates Expected SARSA and eligibility trace-based SARSA(λ) to establish a dual-branch dynamic control framework for isolated intersections. Relying on real-time traffic detection data, a standardized MDP is constructed to realize autonomous signal timing optimization, which can adaptively adjust and switch signal phases according to real-time information such as vehicle queue lengths and travel speeds.

The two branches of the framework are mutually complementary. SARSA(λ) uses eligibility traces for multi-step backpropagation of temporal-difference errors to reuse historical samples efficiently, mitigate learning lag caused by delayed rewards, accelerate policy convergence and cut average vehicle queue length at intersections. Expected SARSA calculates probability-weighted expectations over all subsequent actions instead of single sampled actions, reducing random variance from ε-greedy exploration and restraining Q-value overestimation to stabilize training and lower vehicle waiting delays. Joint optimization of the two branches overcomes high variance, biased estimation and slow convergence of standard SARSA, greatly improving the adaptability of signal control to time-varying random traffic.

All contrast tests are conducted on SUMO-based single-intersection models from four perspectives: convergence, congestion metrics, generalization and hyperparameter stability. Test results show Expected SARSA and SARSA(λ) obtain average Q-values of 99.608 and 99.413, 3.2% and 3.0% higher than baseline SARSA at 96.535. They also converge faster than Rainbow DQN with a more intricate structure. In a standard two-way four-lane case, Expected SARSA shortens average waiting time from 124.94 s to 70.07 s and average queue length from 69.48 m to 44.50 m; SARSA(λ) reduces the two indicators to 73.48 s and 39.41 m. Both methods surpass standard SARSA, DQN and Webster fixed-time timing.

Trials on busy six-lane and eight-lane intersections prove the dual-branch framework consistently shortens queues, eases vehicle delays and lifts driving speed under heavy traffic. Their strengths differ clearly: SARSA(λ) works better on queue and waiting time reduction, while Expected SARSA delivers greater speed improvements. The two methods cooperate well and adapt stably to varied lane layouts and traffic flows. Sensitivity tests on learning rate and exploration rate confirm the algorithm resists parameter disturbances and maintains steady real-time signal adjustment. This lightweight framework features low computation and fast response, meeting online signal control demands. It offers an economical, deployable scheme for adaptive timing at urban single intersections and can be applied to actual road environments.

In addition, all simulation experiments in this paper are only conducted on isolated single intersections without considering traffic flow coupling between road networks, which leaves certain limitations in this research. Future research can be expanded and deepened from three perspectives: integrating multi-source real-world vehicle trajectory data to optimize the MDP state space and reward function, so as to construct a model that better conforms to the operational characteristics of real road networks; introducing dedicated phase constraints for pedestrians and non-motor vehicles to build a multi-modal traffic signal cooperative control model for mixed human-vehicle traffic; extending the dual-branch algorithm framework to large-scale road networks with multiple intersections to realize regional coordinated signal optimization and further enhance the ecological validity and global generalization performance of the model.

## Figures and Tables

**Table 1:** Expected SARSA and SARSA(λ) algorithms.

Steps	Expected SARSA and SARSA(λ) Algorithms
**Input**: learning rate α, discount factor γ, exploration parameter ε, trace parameter λ, maximum number of training episodes N, SUMO environment configuration **Output**: trained Q-table, per-episode total reward sequence R, final policy π	
1: Initialize the environment (SUMO simulation, intersection configuration, etc.) 2: Initialize Q(s,a) values arbitrarily (usually to 0), set learning rate α, discount factor γ, exploration parameter ε, Eligibility trace λ, E(s,a)=0 (usually to 0) 3: For each episode, reset the environment to the initial state $s_{0},$ choose an initial action $a_{0}$ based on the current policy 4: For each time step in the episode t, execute action $a_{t}$ in state $s_{t}$ 5: Observe the next state $s_{t+1}$ and reward $r_{t+1},$ choose the next action $a_{t+1}$ 6: Calculate the TD error: $\Delta t=r_{t+1}+\gamma \times Q\left(s_{t+1},a_{t+1}\right)-Q\left(s_{t},a_{t}\right)$ 7: Update eligibility traces for all state-action pairs: $E\left(s_{t+1},a_{t+1}\right)=\gamma \times \lambda \times E\left(s_{t},a_{t}\right)+1$ 8: Update Q-values for all state-action pairs using the SARSA(λ) formula $Q\left(s_{t+1},a_{t+1}\right)=Q\left(s_{t},a_{t}\right)+\alpha \times \Delta t\times E\left(s_{t},a_{t}\right)$ 9: Update eligibility traces: $E\left(s_{t+1},a_{t+1}\right)=\gamma \times \lambda \times E\left(s_{t},a_{t}\right)$ 10: Transition to the next state $s_{t+1}$ and action $a_{t+1}$ 11: If the episode ends, store the total reward and performance metrics 12: Repeat the above steps until the specified number of training episodes is reached or the Q-values converge	

**Table 2:** Hyperparameters for each reinforcement learning algorithm.

RL	*lr*	*γ*	λ	ε	Steps	CPU	GPU
DQN	0.001	0.95	0.95	0.05	5000	Intel(R) 6230R CPU	NVIDIA RTX A5000
Rainbow DQN							
SARSA							
Expected SARSA							
SARSA(λ)							

**Table 3:** All time constraints.

Constraint Item	Value
Minimum Green Time	10 s
Maximum Green Time	50 s
Amber Time	3 s
All-Red Time	2 s

**Table 4:** Average Q-values of reinforcement learning algorithms.

RL	Mean-Q-Value
DQN	39.640
Rainbow DQN	97.322
SARSA	96.535
Expected SARSA	99.608
SARSA(λ)	99.413

**Table 5:** Experimental results of average vehicle queue length, average vehicle waiting time and average vehicle travel speed at the two-way six-lane intersection.

RL	Average Queue Length	Average Waiting Time	Average Travel Speed
SARSA	69.48	124.94	2.016
Expected SARSA	44.50	70.07	3.282
SARSA(λ)	39.41	73.48	3.200

**Table 6:** Experimental results of average vehicle queue length, average vehicle waiting time and average vehicle travel speed at the two-way eight-lane intersection.

RL	Average Queue Length	Average Waiting Time	Average Travel Speed
SARSA	46.77	85.28	2.736
Expected SARSA	38.85	61.66	3.001
SARSA(λ)	35.06	57.21	3.164

**Table 7:** Traffic performance of RL algorithms under different learning rates (ε = 0.05).

Learning Rate Value	Algorithms	Average Waiting Time of Vehicles	Average Queue Length of Vehicles
*lr* = 0.001	SARSA	85.21	95.72
	SARSA(λ)	68.57	62.33
	Expected SARSA	63.89	65.81
*lr* = 0.01	SARSA	112.71	118.49
	SARSA(λ)	76.82	68.23
	Expected SARSA	74.30	71.57
*lr* = 0.1	SARSA	130.55	129.14
	SARSA(λ)	85.20	74.63
	Expected SARSA	83.78	76.32

**Table 8:** Traffic performance of RL algorithms under different exploration rates (*lr* = 0.001).

Exploration Rate Value	Algorithms	Average Waiting Time of Vehicles	Average Queue Length of Vehicles
ε = 0.05	SARSA	85.21	95.72
	SARSA(λ)	68.57	62.33
	Expected SARSA	63.89	65.81
ε = 0.15	SARSA	80.60	83.38
	SARSA(λ)	60.42	57.70
	Expected SARSA	59.29	58.53
ε = 0.25	SARSA	76.31	78.94
	SARSA(λ)	54.81	51.15
	Expected SARSA	53.07	52.45

## Data Availability

The raw data supporting the conclusions of this article will be made available by the authors on request.

## References

[1] Kerner B.S. (2004). Three-phase traffic theory and highway capacity. Phys. A Stat. Mech. Its Appl..

[2] Geroliminis N., Daganzo C.F. (2008). Existence of Urban-Scale Macroscopic Fundamental Diagrams: Some Experimental Findings. Transp. Res. Part B Methodol..

[3] Yue W., Li C., Chen Y., Duan P. (2021). What Is the Root Cause of Congestion in Urban Traffic Networks: Road Infrastructure or Signal Control?. IEEE Trans. Intell. Transp. Syst..

[4] Liu W.T., Zhang C., Fang W. (2025). Vehicle-level fairness-oriented constrained multi-agent reinforcement learning for adaptive traffic signal control. IEEE Trans. Intell. Transp. Syst..

[5] Liu B., Su K., Wang E. (2025). MATLIT: MAT-based cooperative reinforcement learning for urban traffic signal control. IEEE Trans. Intell. Transp. Syst..

[6] Yang T., Fan W.D. (2024). Transit signal priority under connected vehicle environment: Deep reinforcement learning approach. J. Intell. Transp. Syst..

[7] Wang L., Zhang G., Yang Q. (2025). An adaptive traffic signal control scheme with proximal policy optimization based on deep reinforcement learning for a single intersection. Eng. Appl. Artif. Intell..

[8] Fan S., Lu K., Wang Y. (2024). Action masking-based proximal policy optimization with the dual-ring phase structure for adaptive traffic signal control. IEEE Trans. Intell. Transp. Syst..

[9] Liu Q., Guo Y., An Y., Li M. (2025). Joint lane management and signal optimization for mixed autonomy intersections: An analytical approach. Phys. A Stat. Mech. Its Appl..

[10] Noaeen M., Naik A., Goodman L. (2022). Reinforcement learning in urban network traffic signal control: A systematic literature review. Expert Syst. Appl..

[11] Fuad M.R.T., Fernandez E.O., Mukhlish F. (2022). Adaptive deep q-network algorithm with exponential reward mechanism for traffic control in urban intersection networks. Sustainability.

[12] Gheisarnejad M., Sharifzadeh M., Khooban M.H. (2022). Adaptive fuzzy q-learning control design and application to grid-tied nine-level packed e-cell (PEC9) inverter. IEEE Trans. Ind. Electron..

[13] Zamfirache I.A., Precup R.E., Roman R.C. (2022). Reinforcement learning-based control using Q-learning and gravitational search algorithm with experimental validation on a nonlinear servo system. Inf. Sci..

[14] Liu C., Sheng Z., Chen S., Shi H., Ran B. (2023). Longitudinal control of connected and automated vehicles among signalized intersections in mixed traffic flow with deep reinforcement learning approach. Phys. A Stat. Mech. Its Appl..

[15] Fang Z., Sheng Y., Meng Z., Liu Y., Tang J. (2025). Environment Reconstruction and Trajectory Planning for Automated Vehicles Driving Through Signal Intersection. Phys. A Stat. Mech. Its Appl..

[16] Szoke L., Aradi S., Bécsi T. (2023). Traffic signal control with successor feature-based deep reinforcement learning agent. Electronics.

[17] Wang L., Wang Y.X., Li J.K. (2024). Adaptive traffic signal control method based on offline reinforcement learning. Appl. Sci..

[18] Zheng Y., Luo J., Gao H. (2025). Pri-DDQN: Learning adaptive traffic signal control strategy through a hybrid agent. Complex Intell. Syst..

[19] Lv J., Wang Z., Ma J. (2025). Carbon emission reduction in traffic control: A signal timing optimization method based on rainbow DQN. Appl. Sci..

[20] Lu Y., Li C., Yu H., Wang H. (2025). Soft actor-critic based regional traffic signal control in connected environment and its application in priority signal control. J. Intell. Transp. Syst..

[21] Hu H., Lin S., Wang P. (2025). Control of traffic network signals based on deep deterministic policy gradients. Appl. Intell..

[22] Zhang Y., Zhou Y. (2025). Cooperative multi-agent actor-critic approach using adaptive value decomposition and parallel training for traffic network flow control. Neurocomputing.

[23] Zhai Z., Hao R., Cui B. (2025). HGAT and multi-agent RL-based method for multi-intersection traffic signal control. IEEE Trans. Intell. Transp. Syst..

[24] Fu X., Ren Y., Jiang H. (2025). CLlight: Enhancing representation of multi-agent reinforcement learning with contrastive learning for cooperative traffic signal control. Expert Syst. Appl..

[25] Du Y., ShangGuan W., Chai L. (2022). Traffic signal control in mixed traffic environment based on advance decision and reinforcement learning. Transp. Saf. Environ..

[26] Fang Z., Wang S.Q., Qin Y.Z., Zeng M. (2025). Analyzing the Short- and Long-Term Car-Following Behavior in Multiple Factor Coupled Scenarios. Expert Syst. Appl..

[27] Zhang X., He Z., Zhu Y., Huang W. (2025). Coupled vehicle-signal control based on stackelberg game enabled multi-agent reinforcement learning in mixed traffic environment. Phys. A Stat. Mech. Its Appl..

[28] Wang Z., Yang K., Li L. (2023). Traffic signal priority control based on shared experience multi-agent deep reinforcement learning. IET Intell. Transp. Syst..

[29] Li Y., Zhang H., Zhang Y. (2025). Traffic signal and autonomous vehicle control model: An integrated control model for connected autonomous vehicles at traffic-conflicting intersections based on deep reinforcement learning. J. Transp. Eng. Part A Syst..

[30] Qin Y., Luo Q., Wang H. (2025). Markov chain-based capacity modeling for mixed traffic flow with bi-class connected vehicle platoons on minor road at priority intersections. Phys. A Stat. Mech. Its Appl..

[31] Apostol V.A., Sacala I.S., Caruntu C.F., Ross R., Ceapa V. (2026). An IoT-driven digital twin framework for ai-based traffic management systems: Design and evaluation. Sensors.

[32] Shafik A.K., Rakha H.A. (2025). Decentralized cycle-free game-theoretic adaptive traffic signal control: Model enhancement and testing on isolated signalized intersections. Sensors.

[33] Sutton R.S., Barto A.G. (1998). Reinforcement Learning: An Introduction.

[34] Van Seijen H., Van Hasselt H., Whiteson S., Wiering M. A Theoretical and Empirical Analysis of Expected Sarsa. Proceedings of the 2009 IEEE Symposium on Adaptive Dynamic Programming and Reinforcement Learning (ADPRL).

